# Analysis of the HindIII-catalyzed reaction by time-resolved crystallography

**DOI:** 10.1107/S1399004714025188

**Published:** 2015-01-23

**Authors:** Takashi Kawamura, Tomoki Kobayashi, Nobuhisa Watanabe

**Affiliations:** a Synchrotron Radiation Research Center, Nagoya University, Chikusa-ku, Nagoya 464-8603, Japan; b Graduate School of Engineering, Nagoya University, Chikusa-ku, Nagoya 464-8603, Japan

**Keywords:** HindIII, time-resolved crystallography, freeze-trap method

## Abstract

In order to investigate the mechanism of the reaction catalyzed by HindIII, structures of HindIII–DNA complexes with varying durations of soaking time in cryoprotectant buffer containing manganese ions were determined by the freeze-trap method. In the crystal structures of the complexes obtained after soaking for a longer duration, two manganese ions, indicated by relatively higher electron density, are clearly observed at the two metal ion-binding sites in the active site of HindIII. The increase in the electron density of the two metal-ion peaks followed distinct pathways with increasing soaking times, suggesting variation in the binding rate constant for the two metal sites. DNA cleavage is observed when the second manganese ion appears, suggesting that HindIII uses the two-metal-ion mechanism, or alternatively that its reactivity is enhanced by the binding of the second metal ion. In addition, conformational change in a loop near the active site accompanies the catalytic reaction.

## Abstract 2

A time-resolved study using the freeze-trap method elucidates the mechanism of the DNA-cleaving reaction of HindIII.

## 1. Introduction   

The restriction endonuclease (REase) family of enzymes has several members. In particular, type IIP REases have been widely investigated (Pingoud *et al.*, 2014 ▶) because of their potential in biomolecular engineering. For example, the REBASE database catalogues information about REases (Robert *et al.*, 2010 ▶). Structural features of REases contributing to their restrictive sequence specificity are particularly intriguing, and many REases have been studied using X-ray crystallography. Investigations into the mechanism of the reaction catalyzed by REases, the molecular mechanism of the hydrolysis of the phosphodiester bond, have been reported more recently. Previous studies have indicated that divalent metals ions are cofactors in the reaction mechanism; however, elucidation of their exact roles is challenging since mutating amino-acid residues at the binding site can impact other aspects of the reaction. Several co-crystal structures of REases with bound DNA and divalent metal ions, where the reaction has either been suspended (in the presence of calcium ions) or progressed further along the reaction coordinates (cleaved phosphodiester bonds), have been reported (Viadiu & Aggarwal, 1998 ▶; Lukacs *et al.*, 2000 ▶; Horton & Cheng, 2000 ▶; Horton & Perona, 2004 ▶). The number of metal ions retained at the active site in these structures varies; therefore, mechanisms with one metal ion, two metal ions and even three metal ions have independently been proposed (Pingoud *et al.*, 2005 ▶). While kinetic analysis with computer simulations suggests that one metal ion is sufficient for the reaction to progress (Pingoud *et al.*, 2009 ▶; Xie *et al.*, 2008 ▶), it is still unclear whether the divalent metal ions in these multiple sites observed in the crystal structure are critical for the catalytic reaction of REases (Prasannan *et al.*, 2010 ▶; Pingoud *et al.*, 2014 ▶).

We previously reported a crystal structure of a HindIII–DNA complex (Watanabe *et al.*, 2009 ▶). In this study, crystals were dialyzed against a magnesium ion-containing buffer for a week. In the resolved structure, the bound DNA was already cleaved and two divalent-metal ions (manganese and magnesium ions at sites *A* and *B*, respectively) were observed at the active site. The existence of the two metal ions may suggest a two-metal-ion-based mechanism for the reaction catalyzed by HindIII. However, it is unclear whether two metal ions are necessary for the reaction. For example, it can be argued that one of the ions is introduced after the scission reaction.

Time-resolved techniques are typically used to study catalytic mechanisms, and several methods have been developed. Freeze trapping is one such technique. Usually, the reaction is triggered by introducing a small compound into the crystals of the enzyme by soaking. After an appropriate soaking time, the protein crystals are instantly cooled to trap the reaction intermediate. The solved structures, in which the intermediates are trapped along the reaction coordinate, are a source of information that can facilitate discussions with regard to the catalytic mechanism. Mechanistic insights into enzyme-catalyzed reactions gained by freeze trapping have recently been reported (Nakamura *et al.*, 2012 ▶; Hare *et al.*, 2012 ▶). However, in some cases it is not easy to rationalize the solved structures.

In order to investigate the catalytic mechanism of HindIII, time-resolved crystal structure analysis by freeze trapping was performed. Manganese ion (1 m*M*) was used to trigger the reaction catalyzed by HindIII. For this, crystals of a HindIII–DNA complex were introduced into manganese-containing buffer and flash-cooled after soaking times of appropriate lengths. Some type IIP REases are fully activated by a tenfold lower concentration of manganese ion compared with magnesium ion (Pingoud *et al.*, 2009 ▶). Furthermore, manganese ion is expected to be favoured at site *A* in HindIII (Watanabe *et al.*, 2009 ▶). For these reasons, we can use manganese ion at a lower concentration than magnesium ion. With this low concentration, it is expected that site *A* of HindIII is initially occupied by manganese ion. This may facilitate investigation into the catalytic role of the two metal ion-binding sites in HindIII. In addition, manganese ions have more electrons than magnesium ions, and it is easy to distinguish manganese ions from sodium ions in difference Fourier maps (see below). On soaking, electron densities of manganese ions appear as peaks at the two metal ion-binding sites in the active site. The observed results and the insights into the catalytic reaction mechanism of HindIII are discussed.

## 2. Materials and methods   

### 2.1. Protein crystallization   

All reagents used in this study were analytical grade, and unless otherwise noted they were purchased from Wako Pure Chemical Industries, Osaka, Japan. HindIII was prepared as previously reported (Watanabe *et al.*, 2009 ▶), with the following modification to the purification method: the protein was fractionated using ammonium sulfate before further purification by Ni-affinity chromatography. Concentrated HindIII was stored in 10 m*M* Tris–HCl pH 7.5 buffer containing 200 m*M* NaCl and 10%(*v*/*v*) glycerol. The HPLC-purified oligo DNA 5′-GCCAAGCTTGGC-3′ (the cognate HindIII recognition sequence is underlined), purchased from Integrated DNA Technologies (IDT; Coralville, Iowa, USA), was dissolved in sterilized water (2 m*M*) and incubated at 343 K for 10 min to prepare 12-mer double-stranded DNA (dsDNA; 1 m*M*). HindIII and the dsDNA were mixed so that the concentration of HindIII was 6 g l^−1^ and the HindIII dimer:dsDNA molar ratio was 2:3. The HindIII–DNA mixture was used in crystallization by the hanging-drop vapour-diffusion method. A crystal identical to that reported in the previous study could not be obtained for reasons that remain unknown. The mother liquor used in the crystallization experiments was 100 m*M* Tris–HCl pH 7.5 buffer containing 10–15%(*w*/*v*) PEG 3350, 300 m*M* sodium thiocyanate and 10%(*v*/*v*) glycerol. Blade-like crystals (approximately 200 × 50 × 20 µm) were generated within a week.

### 2.2. Data collection and processing   

For the time-resolved study, a cryoprotectant buffer, *i.e.* 100 m*M* Tris–HCl pH 7.5 containing 10–15%(*w*/*v*) PEG 3350, 300 m*M* sodium thiocyanate, 22.5%(*v*/*v*) glycerol and 1 m*M* MnCl_2_, was prepared. HindIII crystals were dipped into the buffer and after 25, 40, 60 and 230 s were picked up using a nylon loop and flash-cooled at 95 K with N_2_ gas. Since there is no method such as optical absorption spectroscopy that can be used to monitor the progress of the reaction, the time intervals were determined using poor crystals with a trial-and-error process mainly monitoring the peak height, or the occupancy, of the metal sites and the structure of the bound dsDNA. Manganese ion-free cryoprotectant was used to represent the 0 s structure. X-ray diffraction data were collected on beamlines BL5A and AR-NE3A at the Photon Factory, Tsukuba, Japan. The data were indexed, integrated and scaled with *HKL*-2000 (Otwinowski & Minor, 1997 ▶). Diffraction data statistics are summarized in Table 1 ▶.

### 2.3. Structure determination   

The scaled intensity data were converted to structure factors with *CTRUNCATE* (French & Wilson, 1978 ▶) from the *CCP*4 suite (Winn *et al.*, 2011 ▶). Initial phases were determined by the molecular-replacement method with *Phaser* (McCoy *et al.*, 2007 ▶). The HindIII protomer, bound DNA and unbound DNA reported in the previous study (PDB entries 2e52 and 3a4k) were used as search models. The initial models were refined with *REFMAC*5 (Murshudov *et al.*, 2011 ▶) from the *CCP*4 suite and *Coot* (Emsley *et al.*, 2010 ▶). The occupancies of manganese ions in the 25, 40 and 60 s structures were determined by iterative refinement cycles of occupancies, coordinates and temperature factors with *phenix.refine* from the *PHENIX* software suite (Adams *et al.*, 2010 ▶). The convergence of these refined occupancies was affirmed using 0.01 or 0.99 as the initial values.

### 2.4. Validations and analyses   

The determined structures were validated with *MolProbity* (Chen *et al.*, 2010 ▶). Model quality statistics are summarized in Table 2 ▶. Electron density maps with *mF*_o_ − *DF*_c_ amplitudes were prepared after simulated annealing with *phenix.refine* and were displayed with *PyMOL* (v.1.6.0.0; Schrödinger).

## 3. Results   

### 3.1. Structure description   

**Figure 1:**
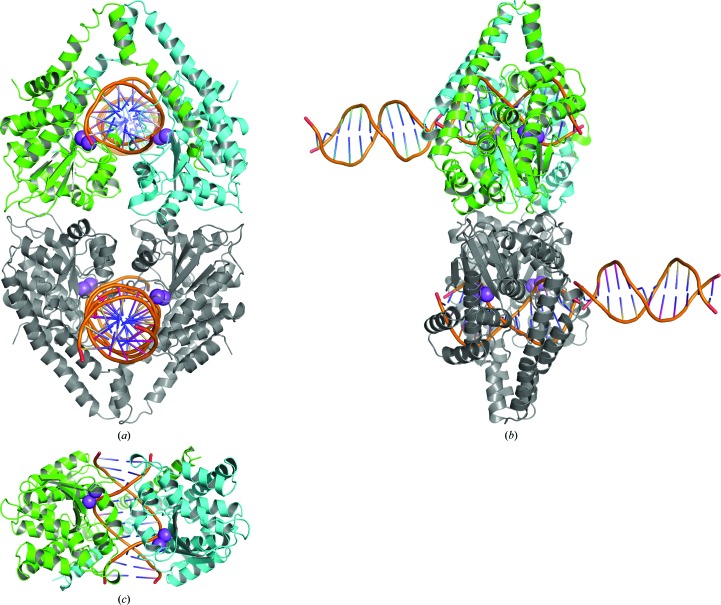
Overall structure of HindIII solved in this study. The HindIII–dsDNA complex and additional dsDNAs in the asymmetric unit are shown in (*a*) and (*b*) (90° rotation). One dimer consisting of two monomers is coloured green and cyan, whereas the other dimer is coloured grey. Each dimer independently binds dsDNA (coloured orange). The biologically relevant complex of the HindIII dimer with dsDNA is shown in (*c*) from a perspective that is rotated 90° around the axis horizontal to the paper from (*a*). Spheres indicate the metal ion-binding sites at the active site.

Crystal structures of the HindIII–DNA complex freeze-trapped 0, 25, 40, 60 and 230 s after dipping into the manganese ion-containing buffer were determined with resolutions of 2.25, 2.54, 2.55, 2.30 and 2.00 Å, respectively. Each crystal structure of HindIII observed in this study has four HindIII polypeptides and eight DNA 12-mer chains; two HindIII dimers are complexed with dsDNA in the asymmetric unit (Figs. 1 ▶ *a* and 1 ▶ *b*). The two HindIII dimers interact with each other, appearing as a dimer of dimers (one dimer is shown as green and cyan ribbons and the other is shown as grey ribbons in Figs. 1 ▶ *a* and 1 ▶ *b*). The observed crystal packing is distinct from that reported previously. Two additional dsDNAs are bound to the dsDNAs, rather than to HindIII, in the HindIII–DNA complexes (orange tubes in Figs. 1 ▶ *a* and 1 ▶ *b*). With these extra dsDNAs, the molar ratio of HindIII dimer:dsDNA in the crystal is 1:2.

The two metal ion-binding sites in the active site of HindIII are shown as purple spheres in Fig. 1 ▶(*c*). In the 0 s structures these sites are occupied by sodium ions. When the crystals are soaked with the manganese ion-containing buffer (25–230 s), growing electron-density peaks, regarded as manganese ions (see below), are observed. Consistent with the catalytic reaction, cleaved products, *i.e.* 4-mer (GCCA) and 8-mer (AGCTTGGC) oligos, are observed in the structure obtained after an immersion time of 230 s. In the 60 s structure both cleaved and uncleaved DNA oligos seem to exist, and models were constructed with half occupancy and superimposed over each other.

Thr117 in all of the solved HindIII structures in this study does not conform to the Ramachandran rules, likely owing to distortions caused by its interactions with the DNA at the major groove. Other validation scores are listed in Table 2 ▶.

### 3.2. Soaking time-dependent structural changes in the active site   

**Figure 2:**
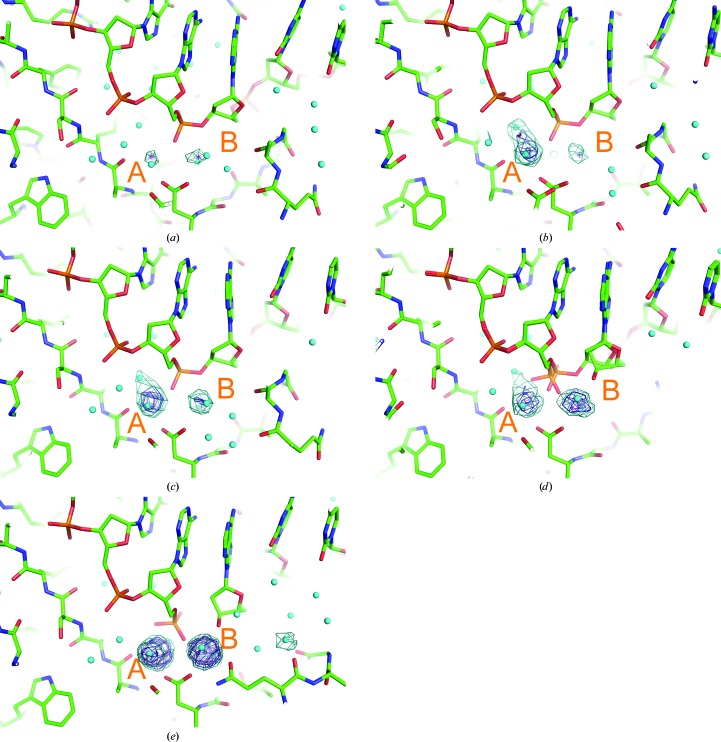
OMIT maps for detecting electron-density peaks of metal ions at metal-binding sites *A* and *B* in the (*a*) 0 s, (*b*) 25 s, (*c*) 40 s, (*d*) 60 s and (*e*) 230 s structures. Only the two metal ions are omitted for calculations. HindIII and the DNA molecules are shown as stick models. Water molecules are shown as small cyan-coloured balls. Electron-density contours are shown at 6σ (cyan), 9σ (deep blue) and 12σ (purple).

In order to investigate the changes in the structure of the active site depending on the duration of the soaking time (0, 25, 40, 60 and 230 s), *mF*_o_ − *DF*_c_ maps omitting ions at the metal-binding sites were calculated. With increases in the duration of soaking time in the cryoprotectant, the increasing presence of manganese ions at the active site of HindIII is indicated by two increasing electron-density peaks (shown as mesh in Fig. 2 ▶). The diffusion time of ions and small molecules into protein crystals varies from seconds to hours (Geremia *et al.*, 2006 ▶). However, the diffusion speed of manganese ions into the crystal of HindIII can be considered to be quick enough for this time-resolved experiment, since the peak height of site *A* shows that the occupancy of the manganese ion is already 0.98 at 25 s. The relatively small electron-density peaks observed in the 0 s structure are attributed to sodium ions, because the mother liquor used for crystallization and the cryoprotectant contain a high concentration of sodium ions (>300 m*M*).

The observed increases in the electron density at the two sites followed different trends. At site *A* an increase in electron density is observed in the 25 s and subsequent structures, whereas at site *B* this starts from the 40 s structure. The delayed incrementation of the electron-density peak at site *B* continues until 60 s. However, in the 230 s structure the heights of the two peaks are nearly equal. Similar changes in the electron density, with minor variations in the peak heights, are observed in each active site of the four HindIII enzymes in the asymmetric unit. We attribute the electron-density peaks in the active site at 230 s as depicting complete occupancy by manganese ions, because the temperature factors of the ions are comparable with the values for the surrounding atoms and the electron-density peaks are sufficiently high (>20σ).

### 3.3. Phosphodiester bond cleavage   

**Figure 3:**
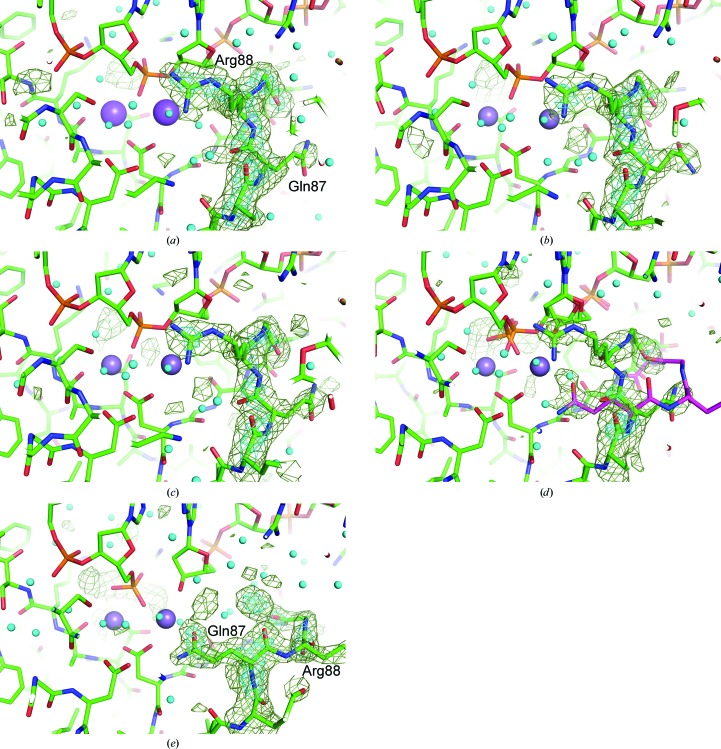
OMIT maps for detecting the cleavage of the phosphodiester bond in the (*a*) 0 s, (*b*) 25 s, (*c*) 40 s, (*d*) 60 s and (*e*) 230 s structures. HindIII and the DNA molecules are shown as stick models. Water molecules are shown as small cyan-coloured balls. Sodium ions in the 0 s structure and manganese ions in structures of the crystals cooled at the other time points are shown as large balls. Electron-density contours are the same as in Fig. 2 ▶. Part of the DNA structure of DA4–DA5 is omitted for the calculations.

The scissile phosphodiester bond of the DNA backbone in the HindIII cognate sequence A/AGCTT was scrutinized using OMIT maps (Fig. 3 ▶). The DNA structure of DA4 and DA5 was omitted from the map calculations. The phosphodiester bond is cleaved at 230 s (Fig. 3 ▶ *e*). At 60 s, however, the electron-density map indicates the presence of a significant amount of uncleaved dsDNA (Fig. 3 ▶ *d*). In structures of the complex prior to 40 s there are no significant conformational changes in the structure of dsDNA. In the 230 s structure, conformational changes directly related to cleavage of the phosphodiester bond between DA4 and DA5 in the DNA are observed.

**Figure 4:**
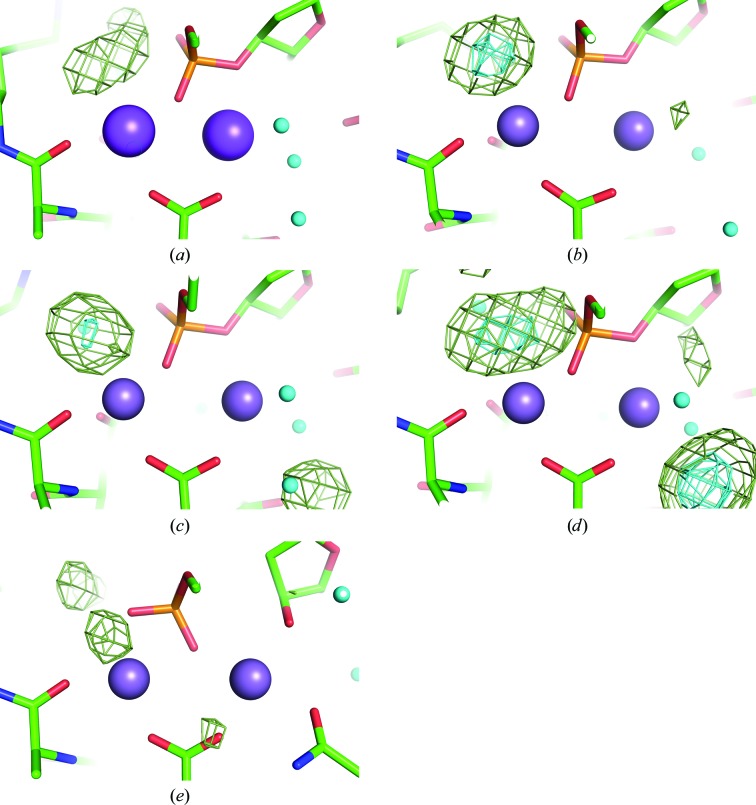
OMIT maps for detecting the electron-density peaks of the nucleophilic water molecule coordinated to the metal ion at site *A* in the structures at (*a*) 0 s, (*b*) 25 s, (*c*) 40 s and (*d*) 60 s. The maps of the 0–60 s structures were prepared with the model of an intact DNA, whereas the 230 s structure was prepared with the cleaved DNA model for phase calculation. The map of the 230 s structure shows that the water molecule has become part of the phosphate group, while remaining coordinated to the manganese ion at site *A*. Electron-density contours are shown at 3σ (green) and 6σ (cyan).

The reaction catalyzed by REases is a nucleophilic substitution reaction with a water molecule as the nucleophile. In the crystal structure of HindIII, a water molecule coordinated to the manganese ion at site *A* is observed as *mF*_o_ − *DF*_c_ peaks in a position poised for attack on the P atom of the DNA backbone (Fig. 4 ▶). Relatively weak electron density representing a water molecule at the equivalent position is observed in the 0 s structure (Fig. 4 ▶ *a*). The electron density at this position increases with duration of immersion time (as in the 25 and 40 s structures; Figs. 4 ▶ *b* and 4 ▶ *c*, respectively). The distorted electron-density peaks observed in the 60 s structure (Fig. 4 ▶ *d*) indicate the formation of the reaction products. In the 230 s structure, electron densities show that the nucleophilic water has become part of the 5′-terminal phosphate moiety of the cleaved DNA (Fig. 4 ▶ *e*).

### 3.4. Loop flipping   

**Figure 5:**
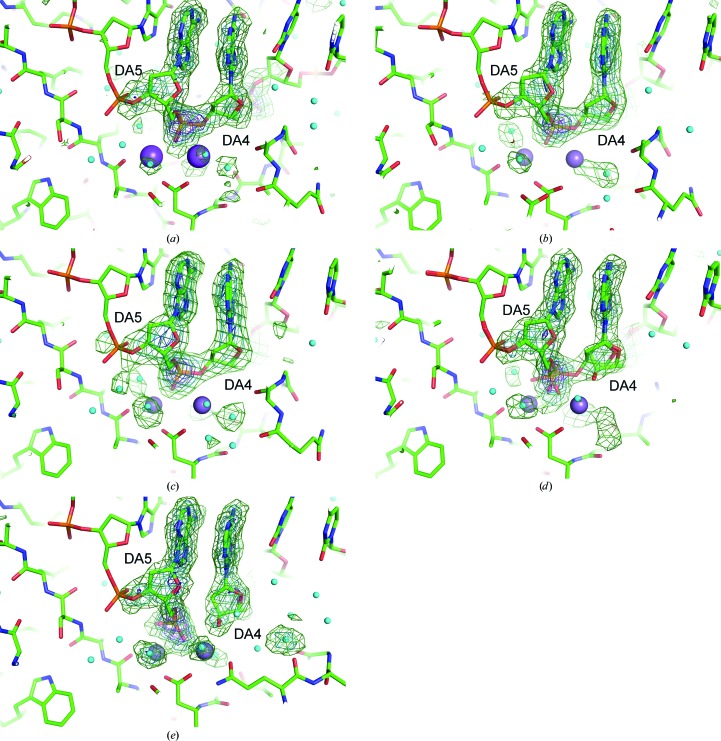
OMIT maps and the interpreted models for the (*a*) 0 s, (*b*) 25 s, (*c*) 40 s, (*d*) 60 s and (*e*) 230 s structures. HindIII and the DNA molecules are shown as stick models. For the loop Gln87–Asn90 in the 60 s structure, alternative conformations were modelled in reference to the 0–40 s (green) and 230 s (magenta) structures. The contour level of these maps is the same as in Fig. 4 ▶.

In the series of structures, a loop near the active site is flipped with the progress of the reaction. This loop, Glu86–Asn90, is located near site *B* and is distant from the nucleophilic water and the scissile phosphodiester bond. When compared with the loop-omitting maps for the 0, 25 and 40 s structures (Figs. 5 ▶ *a*, 5 ▶ *b* and 5 ▶ *c*), the 230 s structure (Fig. 5 ▶ *e*) clearly depicts the flipping of the loop, whereas the loop is only partially flipped in the 60 s structure (Fig. 5 ▶ *d*), resulting in a partially disordered electron-density map. The flipping of the loop results in the rearrangement of two interactions. One of them, the side chain of Arg88, is inserted into the minor groove of the dsDNA and indirectly interacts with the base moiety *via* two water molecules. On flipping, the side chain of Arg88 is expelled from the minor groove and the indirect base interaction is broken. The other interaction involves the side chain of Gln87, which is disordered in the structures of enzyme–DNA complexes with shorter soaking times and is flipped into the active site, coordinating to the manganese ion at site *B*, in the crystal structure with longer soaking time (Fig. 5 ▶ *e*).

## 4. Discussion   

In this study, we have determined time-resolved crystal structures of a HindIII–DNA complex using the freeze-trap method. The reaction was triggered by soaking the crystals with a cryoprotectant containing manganese ion, and the reaction was monitored by varying the soaking time before flash-cooling. Soaking time-dependent variations in the electron-density peaks at the metal ion-binding sites are observed. Phosphodiester bond cleavage in the crystal is also observed.

### 4.1. Metal ion-binding sites   

**Figure 6:**
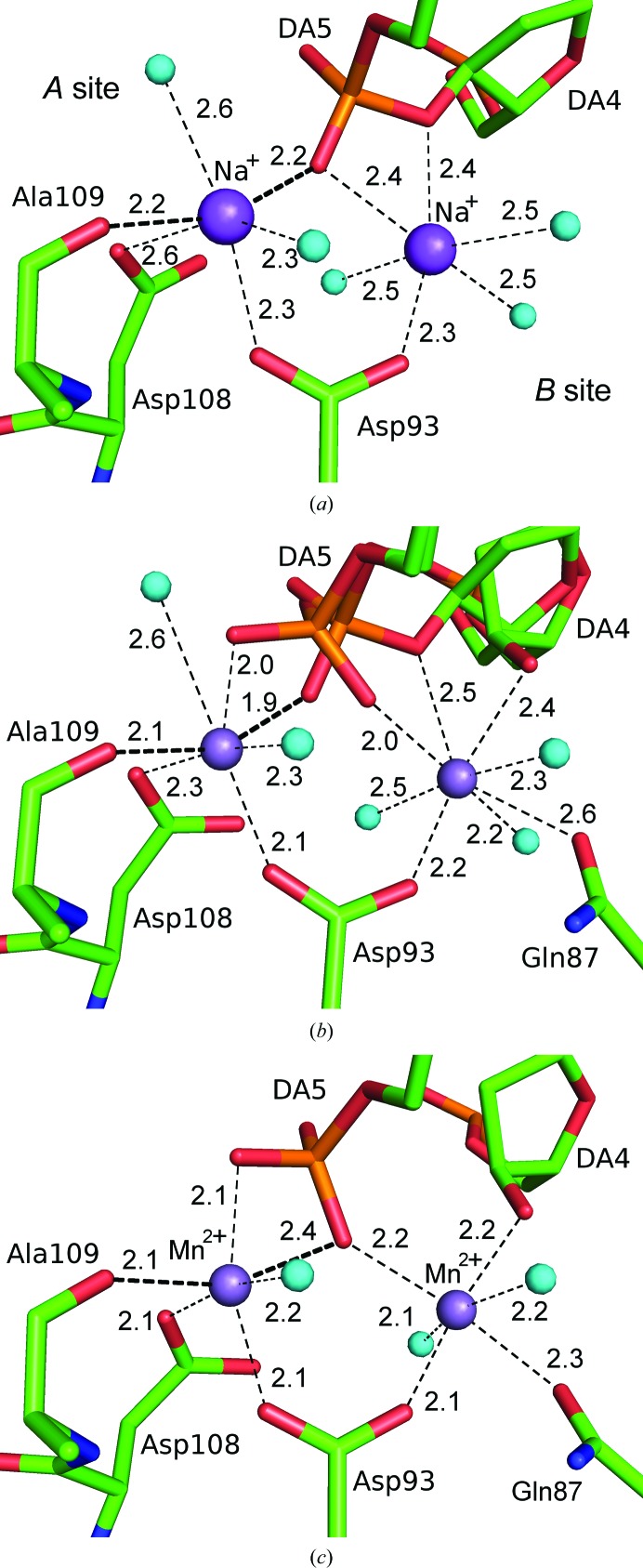
Coordination at the metal ion-binding sites in the (*a*) 0 s, (*b*) 60 s and (*c*) 230 s structures. The coordinate bonds are shown as dashed lines with averaged distances (Å) of the four HindIII molecules in the asymmetric unit. The coordinate bonds from the main-chain O atom of Ala109 and from the phosphate-moiety O atom of DA5 to the Na^+^ ion at site *A* are indicated by bold lines.

In this time-resolved study, electron-density peaks at site *A* increase at a faster rate than at site *B*. At 25 s, the refined occupancies of manganese ion at site *A* have already become almost 1.00; the average for the four subunits is 0.98. Meanwhile, the averaged occupancies of site *B* in the asymmetric unit are 0.47, 0.58, 0.74 and 0.96 at 25, 40, 60 and 230 s, respectively, if the sites were refined as manganese ions. However, the behaviour of these metal sites should be reflected by the increase in manganese ion and the decrease in sodium ion over the soaking time. Since simultaneous refinement of the occupancies and *B* factors of the superimposed ions did not converge, the occupancies of the two metal ions were estimated when refined as manganese. If the sites are occupied by decreasing sodium and increasing manganese ions, the estimated occupancies of manganese ion are 0.06, 0.26, 0.54 and 0.93, while those of sodium ion are 0.94, 0.74, 0.46 and 0.07 at 25, 40, 60 and 230 s, respectively. As shown in Fig. 6 ▶, manganese ion replaces sodium ion and interacts with surrounding side chains at site *A* without inducing significant conformational changes. The coordinations of the metal ion-binding sites are conserved during the catalytic reaction. This suggests that the difference in the rate of occupation by manganese at both sites can be considered as the difference in the intrinsic association and dissociation rate constants of manganese ions and sodium ion between sites *A* and *B*, rather than a sequential binding of manganese ion, such as binding at site *A* inducing the binding of the second ion at site *B*. A possible reason for the difference in the rate constants may result from the difference in the coordination properties at the two sites. At 0 s, the mean distance between the metal and either of the O atoms in the main-chain carbonyl of Ala109 or the phosphate moiety of DA5 is approximately 2.2 Å for the structures in the asymmetric unit (Fig. 6 ▶ *a*). These coordination distances are more suitable for manganese ion (2.15–2.10 Å) than for sodium ion (2.35–2.45 Å) (the typical distances reported are from Hsin *et al.*, 2008 ▶). In the 230 s structure, the manganese ion is clearly coordinated to the two ligands, Ala109 and DA5, with distances similar to those in the 0 s structure, whereas the other coordinate bond lengths are shortened (Fig. 6 ▶ *c*). Furthermore, the two ligands, *i.e.* O atoms from the protein main chain and the DNA backbone, constitute the diagonal of the octahedral coordination at site *A*. Therefore, it is likely that the faster increase in manganese ion at site *A* is derived from the favourable coordination distances to manganese ion than to sodium ion compared with that in site *B*.

### 4.2. Phosphodiester bond cleavage   

The manganese ion adequately occupied site *A* at 25 s, but the averaged occupancy of the manganese ion in site *B* only increased to 0.06. Cleavage of the phosphodiester bond is not yet observed in the electron-density maps. Based on the electron-density maps, the cleavage of the phosphodiester bond is first observed at 60 s, where the occupancy of site *B* becomes 0.54. Therefore, the presence of the manganese ion in site *B* might be a significant factor influencing catalysis by HindIII. For some members of the REase family, a two-metal-ion mechanism has been proposed on the basis of analyses of X-ray crystal structures (Lambert *et al.*, 2008 ▶; Deibert *et al.*, 2000 ▶). Recently, an improved mechanism was proposed in which the ion at site *A* is implicated to be sufficient for catalysis, while the presence of the second ion greatly accelerates the reaction (Xie *et al.*, 2008 ▶). The results of our studies are consistent with this mechanism. Even if the presence of manganese ion at site *A* is sufficient for progress of the reaction catalyzed by HindIII, the cleavage of the phosphodiester bond in DNA with the metal ion only at site *A* is not detected by this freeze-trap approach. The reaction is enhanced by the binding of metal ion at site *B*. A suppressive role of the second ion, as suggested by Pingoud *et al.* (2009 ▶), is not in agreement with the observations made in the case of HindIII. This study reports the experimentally verified role of the two metal ions for the first time.

### 4.3. Conformational shift of the loop at the active site   

We observed a conformational shift of the loop at the active site, a phenomenon that has not previously been observed. However, flexibility of this loop had been predicted from the observed unclear electron density (Watanabe *et al.*, 2009 ▶). The role of this flipping is not obvious; the reason for the trigger of the conformational change, manganese ion binding at site *B* or the phosphodiester bond cleavage, is unclear. However, the interactions that are generated or broken as a result of this flipping are indicative of its roles.

In the course of the conformational change, the side chain of Gln87 is reoriented so as to coordinate with the manganese ion at site *B*. This coordination induces a structural change in the loop, causing the Arg88 residue to flip out from the minor groove of the dsDNA, thereby breaking the interactions between the amino acid and the phosphate backbone of the dsDNA. This loss of hydrophilic and electrostatic interactions between Arg88 and DNA appears to be necessary for the dissociation of HindIII from the cleaved DNA. The two residues Glu/Gln and Arg/Lys are partially conserved in Pfam family RE_HindIII (ID PF09518). Furthermore, previously reported results from activity studies with the E86K mutant, which increases the activity of HindIII (Tang *et al.*, 2000 ▶), suggest the critical nature of the conformational change in the loop for catalysis by HindIII.

## Supplementary Material

PDB reference: HindIII–DNA complex, 3wvg

PDB reference: 3wvh

PDB reference: 3wvi

PDB reference: 3wvk

PDB reference: 3wvp

## Figures and Tables

**Table 1:** Data collection and processing Values in parentheses are for the outer shell.

Soaking time (s)	0	25	40	60	230
PDB code	3wvg	3wvh	3wvi	3wvp	3wvk
Diffraction source	BL-5A	NE-3A	NE-3A	BL-5A	BL-5A
Wavelength ()	1.000				
Temperature (K)	95				
Space group	*P*2_1_				
Unit-cell parameters					
*a* ()	80.25	80.78	80.61	80.50	80.25
*b* ()	141.16	143.29	143.24	140.70	141.16
*c* ()	96.58	94.32	94.36	96.47	96.58
()	112.0	113.5	113.4	112.1	112.0
Resolution range ()	50.02.25 (2.292.25)	50.02.54 (2.592.54)	50.02.55 (2.592.55)	50.02.30 (2.342.30)	50.02.00 (2.032.00)
Total No. of reflections	346596	225118	243043	258376	483264
No. of unique reflections	93316	63629	64086	87848	133005
Completeness (%)	99.7 (97.7)	98.4 (93.8)	99.7 (97.1)	99.9 (100.0)	99.9 (99.8)
Multiplicity	3.7 (3.7)	3.5 (2.9)	3.8 (3.2)	3.8 (3.8)	3.6 (3.4)
*I*/(*I*)	16.5 (2.2)	12.4 (2.9)	14.2 (2.5)	17.0 (2.0)	21.2 (2.1)
*R*_merge_ †	0.128 (0.892)	0.136 (0.591)	0.133 (0.590)	0.154 (0.914)	0.104 (0.804)
Overall *B* factor from Wilson plot (^2^)	31.4	28.9	29.8	34.4	20.0

† *R*_merge_ = 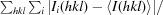 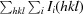.

**Table 2:** Structure solution and refinement Values in parentheses are for the outer shell.

Soaking time (s)	0	25	40	60	230
Resolution range ()	39.72.25 (2.312.25)	32.92.54 (2.612.54)	32.22.55 (2.612.55)	39.72.30 (2.362.30)	39.82.00 (2.062.00)
Completeness (%)	99.6 (97.0)	98.4 (92.1)	99.7 (96.2)	99.7 (97.4)	99.7 (97.1)
No. of reflections, working set	88601	60401	60836	83386	126277
No. of reflections, test set	4714	3227	3249	4430	6688
Final *R*_cryst_	0.180 (0.273)	0.179 (0.284)	0.176 (0.267)	0.184 (0.287)	0.181 (0.275)
Final *R*_free_	0.226 (0.314)	0.241 (0.345)	0.234 (0.349)	0.231 (0.329)	0.226 (0.318)
Cruickshank DPI	0.225	0.463	0.448	0.281	0.145
No. of non-H atoms					
Protein	9808	9808	9808	9808	9808
Na^+^	8	0	0	0	0
Mn^2+^	0	8	8	8	8
Ligand	18	24	24	24	24
Nucleic acid	1944	1944	1944	1946	1948
Water	429	343	378	338	451
Total	12207	12127	12162	12124	12239
R.m.s. deviations					
Bonds ()	0.0159	0.0129	0.0131	0.0139	0.0183
Angles ()	1.7826	1.6111	1.6228	1.6394	1.8840
Average *B* factors (^2^)					
Overall	38.4	30.6	31.4	40.9	36.8
Protein	38.66	30.72	31.94	41.89	36.72
Na^+^	35.86				
Mn^2+^		22.98	28.29	33.80	26.64
Bound dsDNA	24.72	18.40	20.00	29.02	24.52
Extra dsDNA	50.39	43.73	37.66	56.47	51.24
Water	33.73	22.35	24.68	34.18	32.28
Ramachandran plot					
Most favoured (%)	97	96	96	95	97
Allowed (%)	3	3	3	4	3
